# Insomnia: from subjective complaint to physiological signature

**DOI:** 10.1093/sleep/zsaf363

**Published:** 2025-11-11

**Authors:** Wolfgang Ganglberger

**Affiliations:** Department of Neurology, Beth Israel Deaconess Medical Center, Boston, MA, United States; Division of Sleep Medicine, Harvard Medical School, Boston, MA, United States; McCance Center for Brain Health, Massachusetts General Hospital, Boston, MA, United States

Insomnia sits at a tension point in sleep medicine: defined by subjective complaint, yet pursued through objective physiology. Clinicians diagnose it by what patients report, chiefly difficulty sleeping with daytime impairment [1–3]. Yet for decades, researchers have searched the electroencephalogram (EEG) data for an objective “insomnia signature,” with little reward [4–6]. Early polysomnography (PSG) studies revealed only modest and inconsistent differences: people with insomnia slept slightly less and woke more often, but most physiological metrics overlapped with those of good sleepers [7]. Univariate measures such as total sleep time or wake minutes had poor diagnostic power, and even spectral EEG markers yielded conflicting findings. Some studies showed reduced delta and elevated alpha and beta activity, supporting the “hyperarousal” hypothesis [4, 5]. Others found physiologically normal profiles despite severe symptoms, fueling the enduring riddle of “sleep-state misperception” [8]. No single EEG feature could define insomnia. The disorder whispered through many small deviations, if it spoke through physiology at all.

This enduring tension between subjective complaint and objective proof is beginning to ease. The field’s turn toward multivariate and machine-learning approaches is revealing patterns once invisible to traditional analyses. Andrillon et al. (2020) first demonstrated that insomnia leaves subtle yet measurable multivariate EEG signatures: variations in frequency content, spindle dynamics, and spectral slope that, when considered together, differentiated individuals with insomnia from controls—even those whose sleep duration appeared normal [9]. Behind insomnia’s subjectivity lay a physiological substrate discernible only through the integrated pattern of many features.

Pathmanathan et al. (2025) have now brought this multivariate approach to scale. In *Sleep*, they report the development of an Insomnia EEG Score (IES), a machine-learned index trained on more than 3000 overnight PSGs [10]. This represents the largest insomnia EEG dataset assembled to date, comprising roughly 2100 individuals with chronic insomnia and 930 controls. From each recording, the authors extracted over 1300 quantitative features spanning spectral power, spindle dynamics, and macro-architecture.

Baseline separability using demographic variables alone was modest (age, area under the receiver operating characteristic curve (AUC) = 0.62; sex, 0.57). The single best EEG feature—minimum relative delta power across the night—achieved an AUC of 0.74, confirming that no isolated metric suffices to distinguish insomnia from normal sleep. However, performance improved steadily as more features were added: models limited to only a few inputs performed substantially worse, while those allowed to draw on dozens of parameters reached near-maximal discrimination. In other words, insomnia’s EEG signature is distributed, not focal, emerging only when many subtle spectral and structural variations are integrated. The final model incorporated hundreds of features and achieved an AUC of 0.98. Although this figure partly reflects group-definition bias, it nevertheless demonstrates striking separability of insomnia and control recordings based purely on EEG physiology.

The datasets also enabled exploration of insomnia subtypes. Mean IES scores increased progressively across clinical categories: non-insomnia 0.01; subjective-only 0.21; maintenance 0.88; onset 0.95; and combined onset + maintenance 0.996. The graded pattern of IES scores mirrors the gradation of symptoms, indicating that insomnia’s clinical diversity has a measurable physiological correlate.

Together, these results suggest that while insomnia resists reduction to a single feature, its distributed EEG architecture reliably reflects both its presence and its clinical form.

These findings move the field beyond the binary question of whether insomnia has an EEG signature. It does, but that signature is distributed, dimensional, and heterogeneous. The value of this EEG-derived score lies not in diagnostic substitution but in clinical augmentation: stratifying patients, sharpening trial design, and steering treatment choices. Insomnia remains a disorder defined by subjective complaint, yet physiology contributes information the interview cannot capture. The task now is to determine how to integrate that information meaningfully, whether indices such as the IES can identify biological subtypes that respond to specific therapies, or track response and relapse as objective complements to sleep diaries.

Beyond classification, such indices could eventually reshape how insomnia trials are designed and evaluated. Most current studies still rely on relatively simple polysomnographic endpoints, such as wake after sleep onset, sleep latency, or total sleep time; or on subjective measures, including self-reported sleep duration and questionnaires that assess daytime function across sleepiness, mood, and cognitive domains [7, 11–13]. These metrics capture important aspects of the disorder but offer a limited and often divergent view of sleep physiology: perceived total sleep time, for example, can differ markedly from PSG-measured duration in insomnia. A multivariate EEG-based endpoint might provide a richer, more sensitive index of change, integrating hundreds of physiological features into a single measure of sleep quality. Such an approach could increase statistical power, shorten trial timelines, and better connect physiological recovery with patient experience. The next challenge will be to establish whether improvements in such multivariate indices track the outcomes that matter most: how patients feel and function during the day.

Realizing this promise will require careful translation. The IES was trained on datasets drawn from clinical trials, community cohorts, and sleep clinics with varying electrode montages, recording environments, and inclusion criteria. Such heterogeneity strengthens internal validity but complicates generalization. The high accuracy also partly reflects cohort-definition effects: parts of datasets lacked formal DSM-5 insomnia diagnoses, so participants were labeled using a hybrid rule—insomnia if they reported insomnia complaints and had sleep efficiency below 85 percent, versus controls with no complaints and efficiency at or above 85 percent. This made sleep efficiency part of the labeling logic rather than a model feature. In clinical settings, where diagnosis rests on symptoms rather than PSG-defined efficiency, separability may be less pronounced. Metadata were incomplete for key covariates such as medication, caffeine use, and psychiatric comorbidity, each capable of shifting EEG features independently of insomnia. Some apparent misclassifications also reflected plausible physiological alternatives, such as altered delta or alpha activity unrelated to insomnia. Translation will depend on external validation across laboratories, standardized preprocessing, and proof that longitudinal EEG changes track therapeutic improvement.

Future progress will depend on replication and expansion. This study should encourage the creation of additional datasets with more complete metadata and fewer methodological constraints, enabling models that are both more generalizable and more interpretable. Initiatives such as the National Sleep Research Resource [14] and the Human Sleep Project [15] already provide blueprints for large-scale data integration across institutions. Building on and linking these efforts can lay the foundation for a broader, data-driven understanding of the insomnia phenotype.

The EEG-based insomnia score turns a long suspicion into fact: insomnia is not only a complaint but also a physiological state. Those physiological hints became clear only once sufficient scale and analytic depth were available. This advance illustrates how multivariate analysis can relate physiology, behavior, and experience in a single analytic framework. Sleep science, like medicine itself, is moving from isolated measures toward more integrated interpretations of physiology and experience [16–20].

This integrated understanding will strengthen clinical judgment. Multivariate indices can connect observation, mechanism, and decision, providing a shared framework for clinicians, researchers, and those who evaluate medical evidence. Their strength lies in combining many small signals into a more coherent measure. The IES exemplifies how data integration can turn a subjective complaint into a measurable, interpretable dimension of brain health.
